# Artificial Intelligence in Bariatric Surgery: Optimizing Personalized Decision-Making, Predictive Monitoring, and Postoperative Outcomes

**DOI:** 10.1007/s11695-025-08097-9

**Published:** 2025-09-10

**Authors:** Maram Elzayyat, Mohammad Kermansaravi, Jassim Fakhro, Radwan Kassir

**Affiliations:** 1 Qatar University, Doha, Qatar; 2 Hazrat-e Fatemeh Hospital, Tehran, Iran; 3 Hazrat_e Rasool Hospital, Tehran, Ferdows Iran; 4 The View Hospital, Doha, Qatar

**Keywords:** Bariatric surgery, Artificial intelligence (AI), Machine learning (ML), Precision medicine, Predictive analytics, Postoperative complications, Readmission risk, Personalized healthcare, Phenotypic variability, Metabolic surgery, Outcome prediction

## Abstract

Bariatric surgery is an effective treatment for morbid obesity, but patient outcomes differ greatly because of a variety of phenotypes, comorbidities, and postoperative adherence. In bariatric care, artificial intelligence (AI) and machine learning (ML) are becoming revolutionary tools because traditional predictive models based on BMI and demographic variables are unable to account for these complexities. To put it simply, AI is a branch of computer science that enables machines to perform tasks that typically require human intelligence. On the other hand, ML is a subset of AI, where systems learn from data to improve predictions. This study investigates how AI can be used to enhance dynamic, patient-centered follow-up, predict postoperative complications, and improve surgical decision-making. AI can customize interventions, lower complications, and promote long-term weight loss by combining multidimensional data, such as metabolic profiles, behavioral feedback, and phenotypic traits. This study demonstrates how precision powered by AI is laying the groundwork for bariatric surgery in the future.

## Introduction

As a contributing factor to chronic conditions like type II diabetes, cardiovascular diseases, and even sleep apnea, obesity is one of the most serious health issues in the world. For long-term weight loss and metabolic improvement, weight-loss procedures like Roux-en-Y gastric bypass (RYGB) and sleeve gastrectomy have been shown to be the best choices [1, 2]. Nevertheless, variations in body composition, comorbidities, and metabolic responses make a patient’s prognosis extremely unpredictable [1, 3].

Today’s clinical models often work with generalized criteria, relying on BMI thresholds, comorbidity scoring, and subjective evaluation by a medical practitioner [4]. These parameters often miss the multifaceted biological, environmental, and behavioral factors that impact postoperative success [4]. To alleviate the impact of these gaps, more accurate data-based strategies are needed to manage patients around the time of surgery.

Machine learning, a subset of AI, has shown promise in optimizing surgical choices as well as tracking weight loss, predicting postoperative complications and readmission risk following bariatric surgery [5]. Utilizing vast and diverse datasets, AI has the potential to design care plans that meet the specific needs, characteristics, and demographic information of the patients [3, 4].

### The Role of Patient Phenotypes and Demographic Variability in Bariatric Outcomes

The success rates of bariatric surgery are determined by a range of individual-specific characteristics, including body fat distribution, body mass index, basal metabolic rate, and current medical co-morbidities [2]. Patients’ demographics also affect their responses to surgery. Age, social class, race, and sex account for the differences among patients and their participation and compliance with surgical procedures. For example, patients with certain baseline metabolic and nutritional levels, as well as those with certain heredity features, show greatly varying patterns in the amount of weight loss they experience or the risks of complications they face during recovery [1, 2].

Understanding such variability is essential for devising tailored treatment plans. Existing approaches that use singular demographic or BMI information often yield inaccurate predictor models for discernible results [4]. Diverse patient phenotypes point to the need for better predictive functionality. Wu et al. showcases the use of quantitative CT-based body composition metrics in conjunction with other inflammatory and metabolic markers in predicting significant early weight loss post-sleeve gastrectomy, reinforcing the importance of phenotype precision in medical prediction models [2].

Cao et al. demonstrated the significance of patient demographics on postoperative complication rates in a sample of more than 44,000 patients. Their findings revealed that even subtle differences in variables such as waist circumference, HbA1c levels, and comorbidities such as hypertension, diabetes, dyslipidemia, and previous venous thromboembolism significantly influenced outcomes [1].

### AI-driven Precision in Surgical Decision-Making

The personalized approaches enabled through algorithms in machine learning, especially artificial intelligence, are revolutionary in tailoring bariatric procedures to the individual patient [4]. AI algorithms can analyze datasets, which include demographic, phenotypic, metabolic, and even genetic information, to determine what type of procedure, like sleeve gastrectomy or RYGB, will best suit the patient. This kind of data-driven personalization outperforms general guidelines by tailoring treatment to each patient’s unique profile [4].

Wu et al. highlighted the potential to refine the predictive nomogram by incorporating inflammatory profile data along with body fat distribution and insulin sensitivity indices to better forecast outcomes following sleeve gastrectomy or other bariatric procedures [2]. Their approach demonstrates how such models can enhance surgical predictive power. Such advanced models also include age-related metabolic decline, gender-based hormonal fat distribution differences, and other ethnic variations of fat distribution, providing significantly more tailored treatment plans than traditional decision trees [4].

A shift in focus from regression-based approaches to ensemble ML models and deep learning frameworks, which combine multiple learning algorithms to improve accuracy and ultimately reduce errors, as highlighted by Hassan et al. and Mukhtar et al., has proved more efficient for dissecting surgical outcomes of bariatric surgery in multi-center datasets [3, 4].

### Monitoring Weight-Loss Patterns and Patient-Centered Care

Sustained weight loss and early complication detection are both enhanced by careful monitoring after surgery. Nonetheless, postoperative monitoring is either supervised or self-logged and focuses on specific calendar dates, which consistently neglects nutritional deficiencies, behavioral setbacks, and surgical complications for those patients who become lost to follow-up [6].

With the latest advancements in AI, monitoring has become continuous and tailored to each individual through the use of real-time data collected via wearables, mobile devices, and even electronic health records [3, 5, 6]. This constant data collection is often called “streaming data,” which allows AI models to detect subtle patterns as they emerge, rather than relying on static snapshots of health. Recent evidence shows that ML models incorporating such dynamic postoperative data can more accurately predict readmissions than static models using only demographic or clinical information [5]. For instance, Farinella et al. integrates machine learning with dynamic digital follow-up to improve monitoring post-surgery. Their mobile application model that uses a symptom and diet response framework to create alerts had an AUC of 71.5% for identifying those most at risk [6].

This approach denotes vertically integrated healthcare frameworks with patient-centeredness, where responsive care is given. Minor deviations like plateauing or regaining lost weight, which would otherwise be masked by standard timetables, can now be addressed with customized support frameworks [6]. AI has proven useful in turning these possibilities into insights for nutritional counseling, behavioral assistance, and even medication alterations [6].

### Enhancing Outcomes Through Risk Stratification and Complication Management

AI tends to master risk stratification by integrating data from various patient dimensions to predict postoperative complications. Postoperative risks include surgical site infections, gastrointestinal leaks, thromboembolic events, and various cardiometabolic derangements [1, 3]. A study by Cao et al. compared 29 machine learning algorithms and found that ensemble methods like AdaBoost and random forests outperformed traditional logistic regression for predicting severe complications. These models had over 90% sensitivity and specificity in identified datasets [1]. Similarly, Butler et al. demonstrated that ensemble models such as XGBoost and random forest achieved area under the receiver operating characteristic curve (AUROC) scores of 0.785 for readmission prediction, significantly outperforming logistic regression (AUROC 0.62) [5].

Moreover, evolving with continuous learning helps these models adjust risk profiles as new postoperative data becomes available. This is also known as incremental learning, which makes timely, targeted interventions possible while allowing clinical resources to be strategically pooled [3, 4].

Mobile remote monitoring platforms, like the one developed by Farinella et al., further augment these models with perpetual data streams. They capture symptoms like pain, nausea, or suboptimal fluid intake and alert triggered before adverse outcomes occur [6]. Integrating these models allows for a level of safety and responsiveness to foster and develop within postoperative and outpatient settings [3, 6].

### Potential AI Models and Future Directions

More recent systematic reviews like Mukhtar et al. have focused on AI models, especially ensemble methods and neural networks, which are able to predict complications like leaks, infections, readmissions, and nutritional deficiencies with far more accuracy than traditional models [3]. For example, the most impactful predictive variables for readmission include reoperation before discharge, ICU admission, and intraoperative transfusion [5]. That being said, sophisticated models excel at managing “imbalanced data,” where rare outcomes such as severe complications might otherwise be overlooked in predictions. Additionally, they can deal with overfitting issues and population generalization, thereby improving performance across different ethnic groups [3].

Furthermore, Wu et al. stressed the importance of combining inflammatory markers with body composition measurements derived from CT scans for predictive analysis. His model successfully predicted early responders to sleeve gastrectomy, allowing for enhanced, tailored care from clinicians. Consequently, this reinforces the need for phenotype-informed modeling [2].

Collectively, these studies support the acceleration of AI-powered solutions to incorporate patient characteristics such as demographics, phenotypic diversity, comorbidity burden, and streaming data to optimize the refinement and outcomes of precision bariatric surgery.

## Conclusion

Artificial intelligence is redefining bariatric surgery through precision-driven, patient-centered care. From guiding surgical decision-making to monitoring postoperative progress and managing complications, AI offers a scalable, adaptable solution that addresses the complexity and individuality of each bariatric patient. As models continue to evolve and gain external validation, their integration into routine clinical practice holds the promise of improving patient outcomes, reducing complication rates, and enhancing the sustainability of weight loss. With robust data infrastructure, ethical implementation, and interdisciplinary collaboration, AI stands poised to transform the future of obesity treatment.

## Data Availability

No datasets were generated or analysed during the current study.
